# Impact of Fish Oil Supplementation and Interruption of Fructose Ingestion on Glucose and Lipid Homeostasis of Rats Drinking Different Concentrations of Fructose

**DOI:** 10.1155/2017/4378328

**Published:** 2017-08-08

**Authors:** Paola M. Sulis, Katia Motta, Amanda M. Barbosa, Matheus H. Besen, Julia S. da Silva, Everson A. Nunes, Alex Rafacho

**Affiliations:** ^1^Department of Physiological Sciences, Center of Biological Sciences, Federal University of Santa Catarina, 8840-900 Florianópolis, SC, Brazil; ^2^Multicenter Graduate Program in Physiological Sciences, Center of Biological Sciences, Federal University of Santa Catarina, 8840-900 Florianópolis, SC, Brazil

## Abstract

*Background. *Continuous fructose consumption may cause elevation of circulating triacylglycerol. However, how much of this alteration is reverted after the removal of fructose intake is not known. We explored this question and compared the efficacy of this approach with fish oil supplementation.* Methods. *Male Wistar rats were divided into the following groups: control (C), fructose (F) (water intake with 10% or 30% fructose for 9 weeks), fish oil (FO), and fructose/fish oil (FFO). Fish oil was supplemented only for the last 33 days of fructose ingestion. Half of the F group remained for additional 8 weeks without fructose ingestion (FR).* Results. *Fructose ingestion reduced food intake to compensate for the increased energy obtained through water ingestion, independent of fructose concentration. Fish oil supplementation exerted no impact on these parameters, but the removal of fructose from water recovered both ingestion behaviors. Plasma triacylglycerol augmented significantly during the second and third weeks (both fructose groups). Fish oil supplementation did not attenuate the elevation in triacylglycerol caused by fructose intake, but the interruption of sugar consumption normalized this parameter.* Conclusion. *Elevation in triacylglyceridemia may be recovered by removing fructose from diet, suggesting that it is never too late to repair improper dietary habits.

## 1. Introduction

The ingestion of beverages and foods containing a high content of added sugars has been associated with increased prevalence of metabolic disturbances (e.g., dyslipidemias), which may be a risk factor for cardiovascular diseases and diabetes [1, 2]. Fructose is one of the sweeteners intensively used by the food industry (e.g., as high fructose corn syrup) due to its high palatability and the effective low cost [3]. Several rodent models were and still are dedicated to understanding the adverse effects of fructose ingestion on metabolism (by adding fructose in the tap water or in the solid diet) and the most prevalent impacts on metabolism include the elevation of abdominal fat depots [4–8], circulating triacylglycerol levels [4–6, 8, 9] and, although less consistent, the alterations in glucose tolerance [10, 11], insulin sensitivity [12, 13], and basal glycemia [12, 14]. Although there is evidence of a negative effect of fructose per se on glucose and lipid metabolism, these adverse repercussions are more consistent and severe when combined with additional metabolic challenges found in our daily lifestyle, such as the consumption of food containing a high proportion of saturated fat [15, 16]. The impact of fructose ingestion (e.g., in liquid form or associated with a high-fat diet) on cardiometabolic disorders is more deleterious yet when offered in two intercalated periods (e.g., during the prenatal period and adult life) [17, 18]. Considering elevation in plasma lipids has deleterious metabolic and cardiovascular impacts, several studies have investigated the antilipidemic potential of some natural oils (reviewed in Jump et al. 2012 [19] and Zock et al. 2016 [20]). Supplementation with oils containing a high proportion of polyunsaturated fatty acids, especially the ones from the omega-3 family (e.g., fish oils), seemed to be beneficial in contexts of dyslipidemia [19, 20]. A number of experiments with rodents [21, 22] and nonhuman primates [23] demonstrated that supplementation with fish oil has some potential to prevent or attenuate the metabolic alterations caused by continuous fructose or sucrose ingestion. However, translation of doses from rodents to humans, supplementation costs, and lack of continuous adhesion may count against the dissemination of this strategy as an adjuvant therapy for fructose-related adverse effects. Furthermore, the combination of pharmacology and adjuvant therapies (e.g., the use of fish oil supplementation) could improve effectiveness. Refraining from our daily improper habits is a question of counseling and discipline, but if achieved, it could be much more intuitively beneficial than traditional pharmacologic/adjuvant therapies. Based on that, we specifically aimed to investigate the potential benefits of fructose interruption on those parameters altered by early chronic ingestion of fructose. We hypothesized that interruption of fructose ingestion might be more beneficial than supplementation with fish oil.

## 2. Materials and Methods

### 2.1. Ethical Approval

The experiments with rats were approved by the Federal University of Santa Catarina Committee for Ethics in Animal Experimentation (approval ID: PP00782) that is in accordance with the National Council for Animal Experimentation Control (CONCEA).

### 2.2. Materials

Fructose was purchased from LabSynth (Diadema, SP, Brazil). Commercial omega-3 enriched fish oil was purchased from Phytomare® (Governador Celso Ramos, SC, Brazil).  Table 1 details the fatty acids present in the fish oil capsules. Mineral oil was purchased from Farmax (Divinópolis, MG, Brazil). Regular human recombinant insulin (Humulin®) was acquired from Lilly (Indianapolis, IN, USA). The reagents used in the glucose tolerance test, hepatic fat content protocol, fat lipolysis protocol, and histology were acquired from LabSynth and Sigma® (St. Louis, MO, USA).

### 2.3. Animals

Experiments were performed on male Wistar rats accounting for a total of 100 rats. The postweaned rats were obtained from the Federal University of Santa Catarina Animal Breeding Center and were kept at 21 ± 2°C on a 12-hour light–dark cycle (lights on at 0600, lights off at 1800) in conventional laboratory animal breeding. Rats had access to food (commercial standard chow for rats, BIOBASE® 9301, Águas Frias, SC, Brazil) and water ad libitum.

### 2.4. Experimental Design

**Figure 1:**
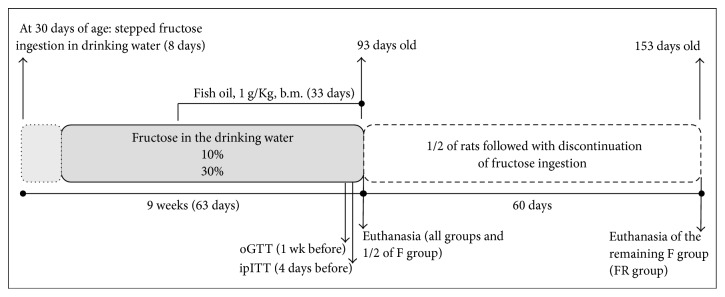
Experimental design. Rats ingested 10% or 30% fructose for 63 consecutive days. Fish oil supplementation was initiated 30 days after the beginning of fructose ingestion and lasted for 33 days until the end of fructose ingestion protocol. Half of the rats on fructose ingestion remained for additional 60 days with discontinuation of fructose (10% or 30%).

According to Figure 1, postweaned rats (21 days of age) were housed for acclimatization for 9 consecutive days and then were randomly separated into five groups in each experimental design.* Experimental design one*: (1) the fructose (F) group (*n* = 20): rats received filtered potable water containing 10% fructose (w/v). Fructose-containing water was offered in a stepped way to avoid abrupt hepatic overload. The fructose (w/v) stepping was 2% for the first 2 days, 4% for the next 2 days, 6% for the next 2 days, and 8% for the next 2 days. From day nine on, rats received water containing 10% fructose (w/v) that was offered for the next weeks accounting for a total of 9-week consumption. (2) The fructose plus fish oil (FFO) group (*n* = 10): rats received fructose-containing water as the F group and one month after the beginning of fructose ingestion rats were supplemented with fish oil (1 g/Kg, body mass (b.m.)) by oral gavage (o.g.), between 0800 and 0900 h, for 33 consecutive days. The dose of fish oil was based on previous publications using rats as an experimental model [24, 25]. The o.g. was interrupted every seven days to simulate an irregular supplementation adhesion (e.g., during one day* per* week). During the 33 days of treatment, a total of 29 supplementations were done. No supplementation was done on the day of euthanasia. (3) The control (C) group (*n* = 10): rats received filtered potable water and mineral oil (1 g/Kg b.m.) by o.g. as a “placebo” of fish oil. No signs of soft feces or diarrhea were observed in these rats. The F group also received mineral oil as the C group. (4) The fish oil (FO) group (*n* = 10): rats received water and fish oil supplementation as the C and FFO groups, respectively. (5) Half of the rats from the F group were euthanized at the end of the fructose ingestion protocol, and the other half (*n* = 10) remained for an additional 60 days with discontinuation of fructose to evaluate the possibility of reversion of those parameters altered by fructose ingestion. This group was named reversibility (FR) group.* Experimental design two*: another set of rats (C: *n* = 10; FO: *n* = 10; F: *n* = 20; FFO: *n* = 10; and FR: *n* = 10 coming from the F group) was studied as the previous experimental design with only one modification as follows: the fructose (w/v) stepping was 5% for the first 2 days, 10% for the next 2 days, 20% for the next 2 days, and 25% for the next 2 days. From day nine on, rats received water containing 30% fructose (w/v) that was offered for the next weeks accounting for a total of 9-week consumption exactly as in the experimental design one. Fish oil supplementation and mineral oil gavage were done exactly as in the experimental design one. The choice of fructose concentrations (10% or 30%) was based on previous studies involving rats for achieving increased levels of circulating triacylglycerol [4, 7, 12].

### 2.5. Metabolic Measurements

Water intake, food intake, and body mass were measured weekly, from the baseline week to the end of protocol accounting for a total of 10 measurements. For the FR group, the measurements occurred every 10 days. Water and food intake were determined by subtracting the cumulative water or food consumed after 24 h from the known amount available a day before and were normalized by the total body mass in each collective cage (five rats per cage). Energy intake was calculated weekly by the sum of chow, fructose, and fish oil calories and normalized by body mass. The proportion of fructose contribution for total calorie intake was calculated according to the following formula: (Kcal from fructose × 100)/(Kcal from chow + Kcal from fructose + Kcal from fish oil, when it was the case). The growth rate was calculated by the following formula: (final b.m. − initial b.m.)/initial b.m. × 100. Blood glucose and plasma triacylglycerol were measured in fasted (12–14 h) rats every 10 days, from the baseline day to day 60, for a total of 7 measurements. These analyses were also done in the FR group every 10 days. During fasting analyses, the fructose-containing water bottles were replaced by tap water without fructose. Blood was collected from the tail to measure blood glucose levels with a glucometer (Accu-Check® Performa, Roche Diagnostics GmbH, Mannhein, Germany) and, thereafter, 80 *μ*l of blood was sampled for posterior plasma triacylglycerol quantification. Blood was collected in EDTA-NaF-containing tubes (Glistab–Labtest, Lagoa Santa, MG, Brazil) to obtain the plasma (600 ×g centrifugation), which was stored at −80° until triacylglycerol determination.

### 2.6. Oral Glucose Tolerance Tests (oGTT)

The oral glucose tolerance test was performed 1 week before the day of euthanasia (Figure 1). Fasted (12–14 h) rats had their tail tip cut for blood collection. The first drop was discarded, and the second drop was used for the determination of glycemia (time 0) using a glucometer as described before. Then, a 50% glucose solution prewarmed at 36°C (2 g/Kg, b.m., o.g.) was immediately administered, and blood samples were collected from the tail tip at 30, 60, and 120 min for blood glucose measurements. The area under curve (AUC) to blood glucose values was performed for each animal with normalization of data by the lower glycemic value [26, 27].

### 2.7. Insulin Tolerance Test (ITT)

The insulin tolerance test was performed 4 days before the day of euthanasia (Figure 1) in fed rats that had blood collected and glycemia measured (time 0) as for oGTT. An insulin solution prewarmed at 36°C (2 IU/Kg, b.m., i.p.) was immediately administered, and blood samples were collected from the tail tip at 10, 20, and 40 min for blood glucose measurements. The constant rate of glucose disappearance (*K*_ITT_) was calculated from the slope of the regression line obtained with log-transformed glucose values between 0 and 40 min after insulin administration [26, 28].

### 2.8. Euthanasia and Biochemical Data

Rats were euthanized by exposure to CO_2_ followed by decapitation, and the trunk blood was collected into EDTA-NaF-containing tubes (Glistab–Labtest) to obtain the plasma and be stored as described above. Plasma insulin was quantified by AlphaLISA® technology (Perkin Elmer, Waltham, MA, USA, cat. number AL204), according to manufacturer's instructions. Enzymatic colorimetric assays for the quantification of plasma triacylglycerol and uric acid were from Biotécnica (Varginha, MG, Brazil) and the quantification was done according to the manufacturer's instructions. Organs (listed in Table 2) were gently withdrawn and weighed. Visceral fat was composed of omental, retroperitoneal, and epididymal depots.

### 2.9. Determination of Insulin Sensitivity by Triacylglycerol and Glucose Index (TyG)

TyG index was calculated using the following formula: Ln[fasting triacylglyceridemia (mg/dL) × fasting glycemia (mg/dL)/2] as previously published [29].

### 2.10. Hepatic Triacylglycerol Content

Triacylglycerol content: determination of hepatic triacylglycerol content was in accordance with Gonçalves-Neto and colleagues [30]. Briefly, liver samples were transferred to test tubes containing 0.7 mL 1 M NaCl and homogenized with T18 UltraTurrax® (IKA®, Staufen, Germany). Then, methanol/chloroform solution (1 : 2 v/v) was added, and the tubes were subsequently centrifuged. The methanolic phase was then transferred to another test tube, and after drying the solvent in hot water, a solution of methanol/Triton 100 (1 : 2 v/v) was added to the samples for determination of hepatic triacylglycerol content that was normalized by the tissue fragment mass.

### 2.11. Liver Histology and Adipocyte Size

To study the morphological aspects of the liver, a minimum of five liver fragments (the same portion for all) from each group was excised and immersion-fixed for 24 h in 4% paraformaldehyde fixative solution, dehydrated, and embedded in paraffin. The largest liver area in the block was cut (5 *μ*m) on a rotary microtome and adhered to individual regular glass slides. Fragments of epididymal adipose tissue (the same portion for all animals) were processed as for liver fragments. Liver and epididymal sections were stained with Hematoxylin & Eosin to perform the morphological and morphometrical analyses, respectively, and the images were then registered by a CCD camera coupled to a BX-60 Olympus photomicroscope [30]. The mean adipocyte size, based on adipocytes area (in *μ*m^2^) of epididymal sections, was obtained from a total of 1,800 cells (300 random cells × 6 random sections per each experimental group, each section being derived from a different rat). Liver and adipose tissue histology were performed only in the experimental design with 30% fructose ingestion.

### 2.12. Adipose Tissue Lipolysis

Adipose tissue lipolysis was performed immediately after the euthanasia according to previous publications [31, 32]. The ex vivo adipose tissue lipolysis was assayed by incubating tissue samples (the same portion for all animals) and evaluating glycerol release into the incubation medium. Epididymal adipose tissue fragments (100 mg) were incubated in aerated (5% CO_2_ : 95% O_2_) Krebs buffer (pH 7.4) containing 1% bovine serum albumin for 60 min at 37°C in the presence or absence of 20 *μ*mol/L isoproterenol. At the end of the incubation, samples were collected and kept on ice. Glycerol concentrations were determined by an enzymatic colorimetric assay, similar to that described for plasma triacylglycerol.

### 2.13. High-Performance Liquid Chromatography (HPLC)

HPLC was used to determine fatty acids in the commercial fish oil. Phospholipids, triacylglycerol, cholesterol esters, and free fatty acids were extracted using chloroform : methanol (2 : 1, v/v), using the method described by Folch et al. (1957) [33]. Saponification proceeded in methanol with the pH adjusted to ≥12 with 5 mol/L NaOH. The pH was adjusted to <3 and the sample was subjected to a new lipid extraction by hexane followed by evaporation in gas N_2_ at 37°C. Fatty acids were derivatized with 4-bromomethyl-7-coumarin and acetonitrile [34] and subsequently separated into a reversed phase analytical column brand Sigma, MV-C8 4.6 mm 25 cm idx particles of 5 microns (Supelco®). Sample analysis was performed with a Waters Alliance Separation Module e2695 (Waters, Milford, MA, USA). 1.6 *μ*L of diluted derivatives was injected and then eluted isocratically by the binary gradient of water and acetonitrile (70–30%) at 80 min run between 18 and 21°C. The compounds were detected fluorometrically (multifluorescence detector 2475, Waters), with excitation at 325 nm and emission at 398 nm. Data were recorded and integrated by Empower Pro Version 2.0 software. A standard curve containing the following fatty acids was applied for the relative quantification: myristic, lauric, palmitic, palmitoleic, oleic, stearic linoleic, alpha-linolenic, arachidonic, eicosapentaenoic, and docosahexaenoic acid. Data were expressed as a percentage of individual fatty acids.

### 2.14. Statistical Analysis

The results are expressed as the mean ± SEM of the indicated number (*n*) of animals. The symmetry of the data was tested by Kolmogorov-Smirnov and Shapiro-Wilk normality tests. Analysis of variance (ANOVA) (one-way ANOVA) for unpaired groups followed by Tukey's post hoc test was utilized for multiple comparisons of parametric data. Kruskal-Wallis followed by Dunn's post hoc was used when variables achieved asymmetric distribution. When indicated, Student's* t*-test and Mann-Whitney for unpaired data were also applied. Extreme studentized deviate method was applied to determinate whether one of the values reached significant outlier (Grubb's test from online available GraphPad QuickCalcs). Significance was set at *p* < 0.05.

## 3. Results

### 3.1. Rats Drinking Fructose-Containing Water Did Not Alter the Overall Energy Intake Independent of Fructose Concentration in the Water

**Figure 2:**
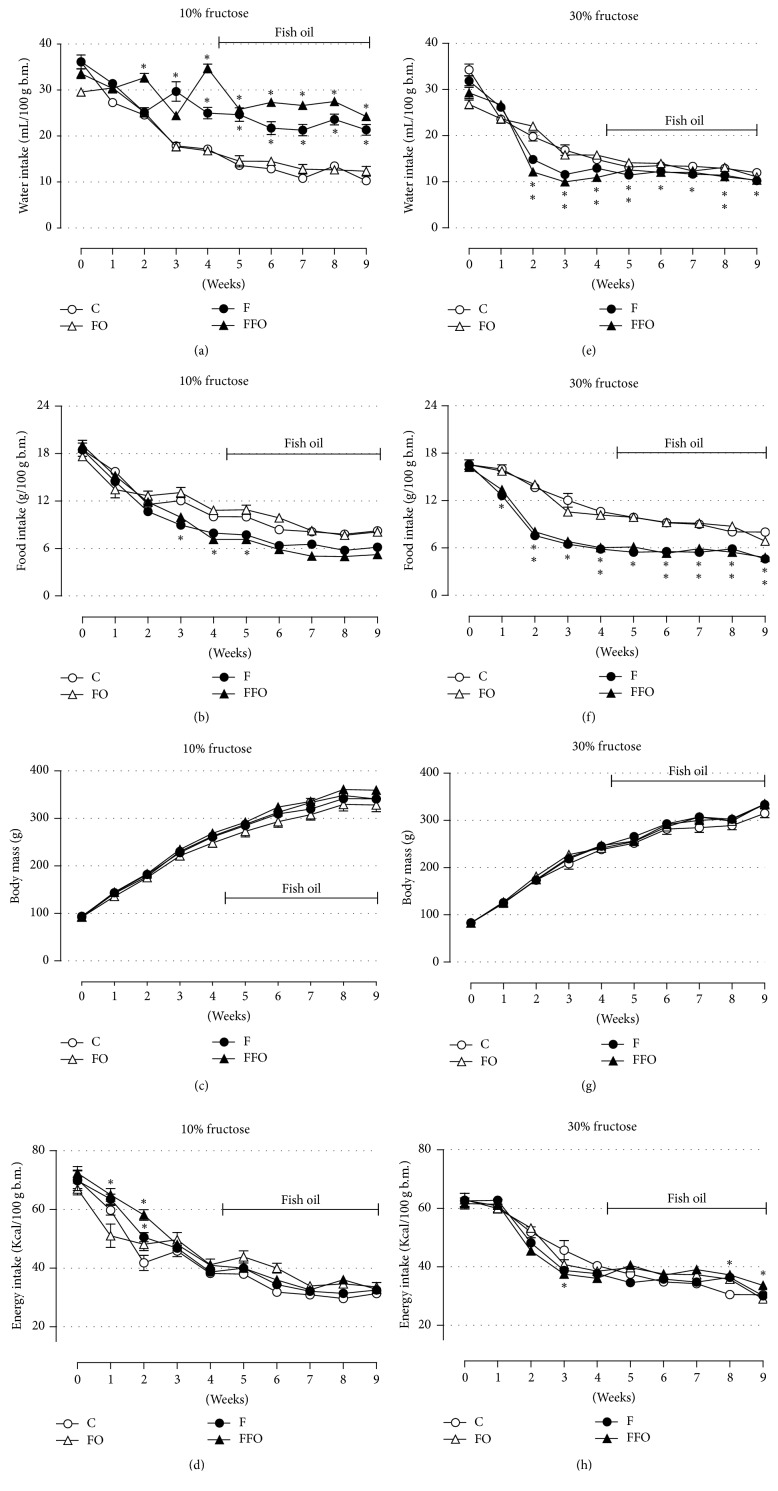
Chronic fructose ingestion was not effective in altering body mass. The average water intake (a, e), food intake (b, f), b.m. (c, g), and energy intake (d, h) in rats drinking water enriched with 10% fructose (w/v) (a–d) and 30% fructose (w/v) (e–h). Fructose ingestion lasted 9 consecutive weeks, and fish oil supplementation (1 g/Kg, b.m.) was introduced 30 days after the initiation of fructose ingestion and remained until the end of the ninth week. Results are expressed as mean ± SEM. Asterisk (*∗*) indicates significant difference compared with the respective control groups (the C and FO groups) using ANOVA with Tukey's post hoc test (*p* < 0.05, *n* = 10–20). b.m.: body mass.

No differences in the water and food intake were observed among the 4 groups, in either the* b.m.* or energy intake, in each experimental design, before initiation of the treatments (Figures 2(a)–2(h) and Supp. Figure  1(a) in Supplementary Material available online at https://doi.org/10.1155/2017/4378328). The major aspects observed during ingestion of water containing 10% fructose were a higher intake of water (versus the C group) that parallels a reduction in the food intake (versus the C group), reaching statistical significance in the second and third weeks, respectively, until the end of the ninth week (Figures 2(a) and 2(b) and Supp. Figure  1(a)) (*n* = 10–20; *p* < 0.05). A similar trend was observed for the food intake in rats drinking water enriched with 30% fructose, which ingested lower volumes of water in relation to the control group (Figures 2(e) and 2(f)) (*n* = 10–20; *p* < 0.05). The b.m., including growth rate, and overall energy intake were similar among the four groups along the 9 weeks in each experimental design (Figures 2(c), 2(d), 2(g), and 2(h) and Supp. Figures  1(b), 1(c)), except for an increase in energy intake during weeks 1 and 2 (for the F group in 10% fructose panel) and weeks 8 and 9 (for the F group in 30% fructose panel). This energy intake increase did not influence the b.m. during the experiment. Fish oil supplementation exerted no significant impact on any of the parameters evaluated (Figures 2(a)–2(h) and Suppl. Figures  1(a)–1(c)). Overall, fructose ingestion, independent of the concentration, was not effective in causing increased b.m.

### 3.2. Concentration-Related Contribution of Fructose to Energy Intake

**Figure 3:**
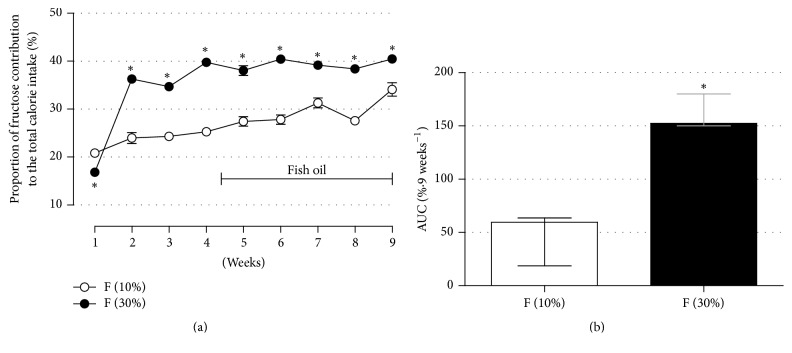
Proportion of fructose contribution to the total calorie intake. The average proportion values of fructose contribution to the total amount of caloric intake during experimental protocol (a) and the AUC for such proportion values (b). Fructose ingestion lasted 9 consecutive weeks, and fish oil supplementation (1 g/Kg, b.m.) was introduced 30 days after the initiation of fructose ingestion, remaining until the end of the ninth week. Results are expressed as mean ± SEM for (a) and median ± interquartile range for (b). Asterisk (*∗*) indicates significant difference compared with the F (10%) group using Student's* t*-test in (a) and Mann-Whitney in (b) for unpaired data (*p* < 0.05, *n* = 30).

To evaluate the contribution of fructose to the overall caloric intake, we calculated the proportion of fructose to energy. Although rats from the F groups (both 10% and 30%) ingested the same amount of energy per b.m. (that were similar to C and FO groups), the contribution of fructose as total energy consumed was higher in the rats from the 30% fructose group (Figures 3(a) and 3(b)) (*n* = 30; *p* < 0.05). During the first week, the proportion of calories from fructose in the total energy consumed was higher in rats from the 10% fructose group, but this relationship was inverted after the second week and stayed significantly higher for the next 8 weeks of treatment in the 30% fructose group.

### 3.3. Fish Oil Supplementation Has No Positive Impact on Elevated Circulating Triacylglycerol Caused by Fructose Ingestion

**Figure 4:**
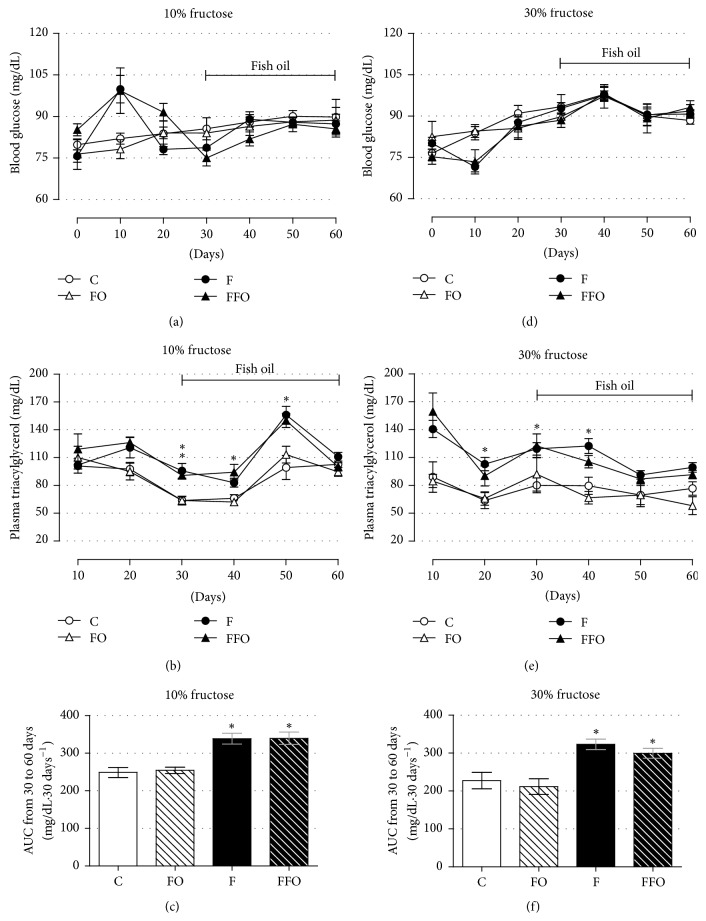
Fish oil supplementation did not improve the circulating triacylglycerol levels in rats drinking fructose. The average blood glucose (a, d), plasma triacylglycerol (b, e), and AUC (c, f) in rats drinking water enriched with 10% fructose (w/v) (a–c) and 30% fructose (w/v) (d–f). Fructose ingestion lasted 9 consecutive weeks, and fish oil supplementation (1 g/Kg, b.m.) was introduced 30 days after the initiation of fructose ingestion and remained until the end of the ninth week. Results are expressed as mean ± SEM. Asterisk (*∗*) indicates significant difference compared with the respective control groups (the C and FO groups) using ANOVA with Tukey's post hoc test (*p* < 0.05, *n* = 10–20). AUC: area under curve.

Fructose ingestion, at both 10% and 30% concentration, caused no significant impact on blood glucose values during the 60-day evaluation (Figures 4(a) and 4(d)) (*n* = 10–20). Consumption of water containing 10% and 30% fructose resulted in elevation of circulating triacylglycerol levels, being significantly different from control animals at days 30 and 50 for the F group (10% fructose panel) and at days 20, 30, and 40 for the F group (30% fructose panel) (Figures 4(b) and 4(e)) (*n* = 10–20; *p* < 0.05). No significant differences were observed in the last day evaluated (day 60) independent of the percentage of fructose consumed. The AUC, calculated from the beginning to end of fish oil supplementation (days 30 to 60), revealed no major effects of fish oil supplementation on such parameter (Figures 4(b), 4(c), 4(e), and 4(f)) (*n* = 10–20). Fish oil supplementation per se had no impact on such parameters. Thus, regular fructose intake led to a moderate and transitory elevation in circulating triacylglycerol levels. Nevertheless, fish oil supplementation had no impact on triacylglyceridemia.

### 3.4. Chronic Ingestion of Fructose-Containing Water Has No Major Impact on Glucose Tolerance and Insulin Sensitivity

**Figure 5:**
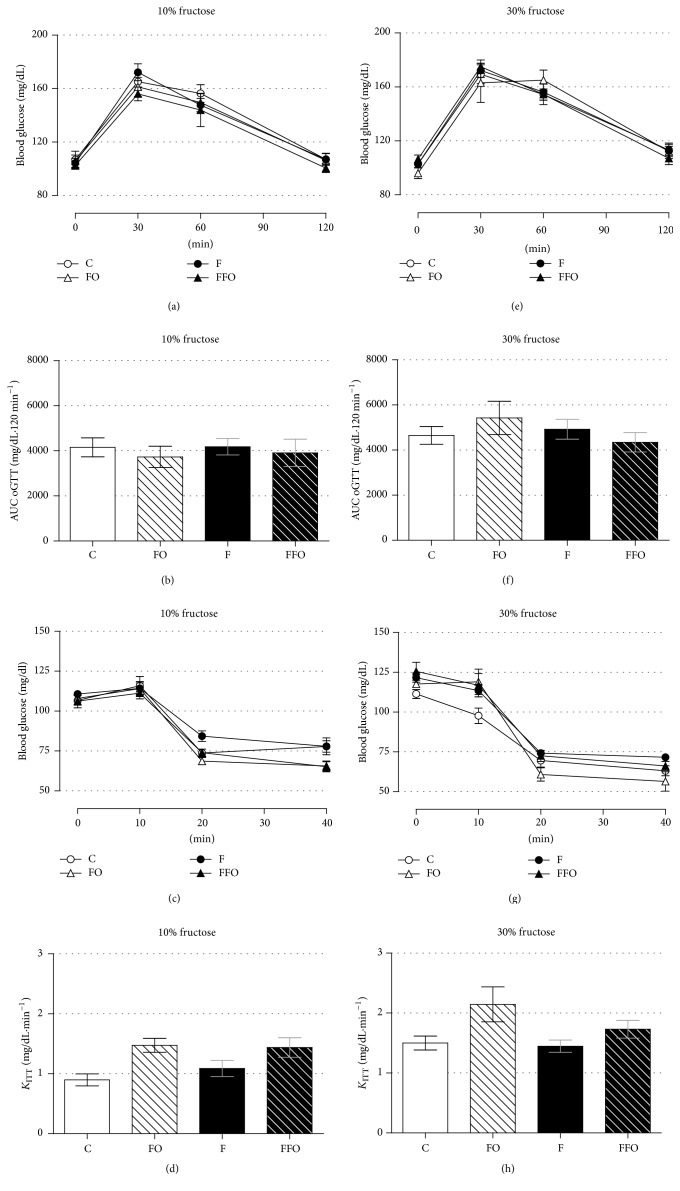
Chronic fructose ingestion did not alter glucose tolerance or insulin sensitivity, and fish oil supplementation had no effect per se. The average blood glucose values during an oGTT (a, e), AUC for blood glucose values (b, f), blood glucose values during an ipITT (c, g), and *K*_ITT_ (from 0 to 40 min) (d, h) in rats drinking water enriched with 10% fructose (w/v) (a–d) and 30% fructose (w/v) (e–h). Fructose ingestion lasted 9 consecutive weeks, and fish oil supplementation (1 g/Kg, b.m.) was introduced 30 days after the initiation of fructose ingestion and remained until the end of the ninth week. Results are expressed as mean ± SEM. ANOVA with Tukey's post hoc test was applied and no differences among groups were achieved (*n* = 10–20). AUC: area under curve; *K*_ITT_: constant for glucose decay.

Both glucose tolerance (based on the oGTT) and insulin sensitivity (based on ipITT) remained unaltered in the F groups of rats (10% and 30% panels) compared to the C groups (Figures 5(a), 5(c), 5(e), and 5(g)) (*n* = 10–20). The AUC and *K*_ITT_ calculi reinforced this (Figures 5(b), 5(d), 5(f), and 5(h)) (*n* = 10–20). Again, supplementation with fish oil (the FO and FFO groups) for 26 and 29 days (until oGTT and ipITT experiments, resp.) had no impact on these parameters (Figures 5(a)–5(h)) (*n* = 10–20). Altogether, these data demonstrate that continuous fructose ingestion (approximately 60 consecutive days), independent of 10% or 30% concentration, did not cause a deleterious impact on glucose tolerance and insulin sensitivity in male adult rats.

### 3.5. The Increase in Visceral Fat Depots Depends on the Proportion of Fructose Intake

Although fructose intake led to no major metabolic alterations, we evaluated a number of variables immediately after the euthanasia as follows. Sixty-three days of fructose ingestion resulted in hyperinsulinemia (2.28-fold increase in the F group drinking water with 10% fructose and 4.61-fold increase in the F group drinking water with 30% fructose) (Table 2) (*n* = 8–10; *p* < 0.05). The FFO groups (10% and 30% panels) had similar hyperinsulinemia when compared to the F group. Urecemia, hepatic triacylglycerol content, and basal or stimulated glycerol release (from epididymal fat fragments) were altered by neither the fructose intake nor the fish oil supplementation (Table 2) (*n* = 10–20). The liver histology, evaluated only in the experimental design with 30% fructose ingestion, revealed no major impact of fructose ingestion, except for signs of fat accumulation in 1 out of 10 animals analyzed (Supp. Figure  2). The relative visceral fat mass (a sum of omental, epididymal, and retroperitoneal depots) and TyG index (a predictor of altered insulin sensitivity) were augmented in the F group (only 30% fructose) compared to the C group (Table 2) (*n* = 10–20; *p* < 0.05). This increased relative visceral adiposity in the F group (only 30% fructose) was not associated with reduced or increased adipocyte area that could suggest adipocyte hyperplasia or hypertrophy, respectively (Supp. Figures  3(a)–3(c); *n* = 6). When evaluated individually, none of the relative visceral masses (omental, retroperitoneal, and epididymal) were significantly increased in the F group (data not shown). Fish oil supplementation did not improve such altered parameters and had no effect per se (*n* = 10–20). Overall, these data indicate that an accumulation of visceral fat mass depends on the proportion of fructose intake and this is associated with markers of reduced insulin sensitivity.

### 3.6. Interruption of Fructose Ingestion Contributes to an Improvement of Metabolic Parameters Altered by Sugar Consumption

**Figure 6:**
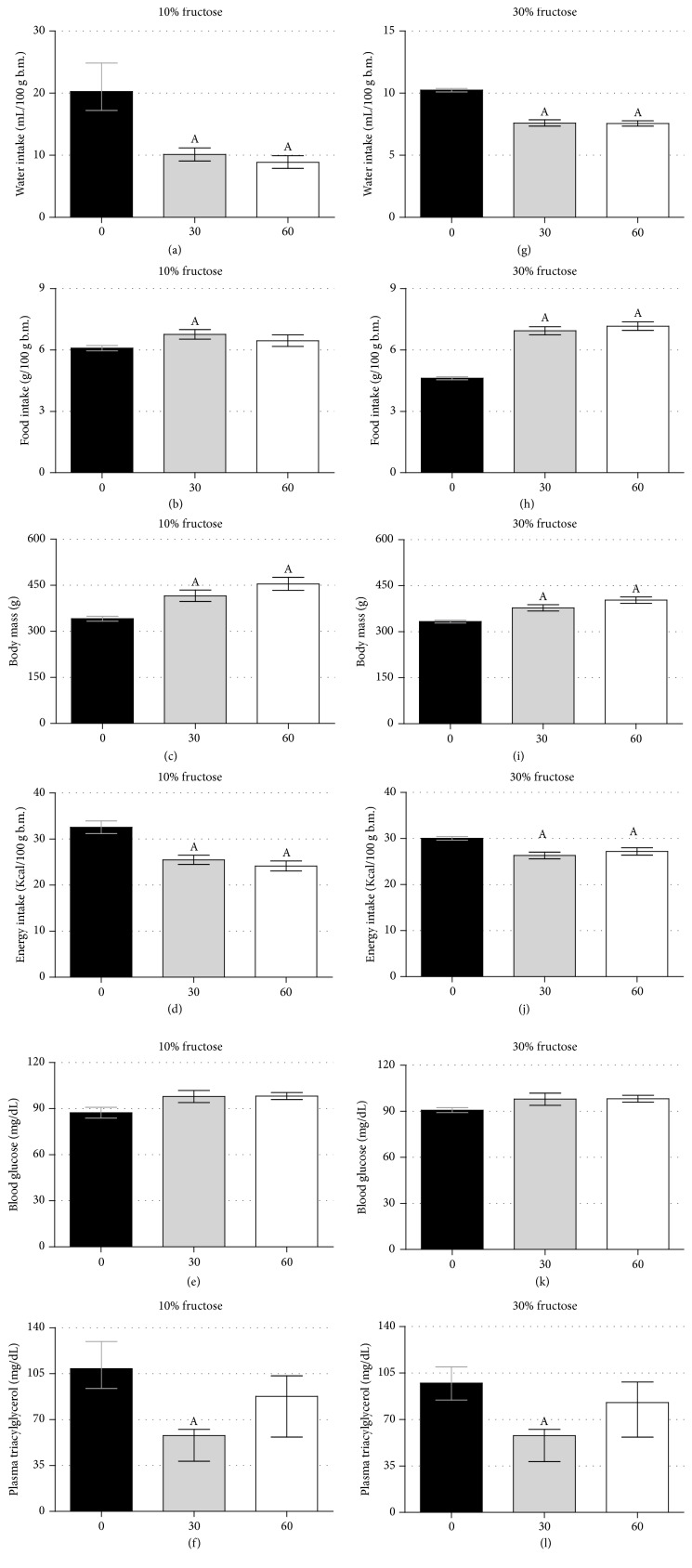
Interruption of fructose ingestion resulted in normalization of water and food intake behavior. The average water intake (a, g), food intake (b, h), b.m. (c, i), energy intake (d, j), blood glucose (e, k), and plasma triacylglycerol (f, l) in rats drinking water enriched with 10% fructose (w/v) (a–f) and 30% fructose (w/v) (g–l). Fructose ingestion lasted 9 consecutive weeks, and it was considered day 0 for this set of data. Then, fructose was discontinued in the water, and half of the rats from the F group remained for additional 60 days (the other half was euthanized at the end of the fructose intake protocol). Results are expressed as mean ± SEM for (a–e) and (g–k) and median ± interquartile range for (f, l). Letter A indicates significant difference compared to day 0 using ANOVA with Tukey's post hoc test for (a–e) and (g–k) and Kruskal-Wallis with Dunn's post hoc test for (f, l) (*p* < 0.05, *n* = 20 at baseline “day 0” and *n* = 10 at days 30 and 60). b.m.: body mass.

We then sought to evaluate how the organism could respond to discontinuation of fructose ingestion. As observed in Figure 6, we measured 6 parameters at 30 and 60 days after the interruption of fructose ingestion and the results were quite similar, independent of the fructose regimen used (10% and 30%). Water and food intake were normalized after discontinuation with rats reducing the amount of water intake and increasing the amount of food intake (Figures 6(a), 6(b), 6(g), and 6(h)) (*n* = 20 at baseline “day 0” and *n* = 10 at days 30 and 60; *p* < 0.05). This reversion of water and food intake patterns resulted in a reduced energy intake (Figures 6(d) and 6(j)) and the expected normal body weight mass increased with the continuation of animal growth (Figures 6(c) and 6(i)) (*p* < 0.05). The blood glucose values remained unaltered at 30 and 60 days after fructose discontinuation (*p* < 0.05) (Figures 6(e) and 6(k)). The circulating triacylglycerol levels were reduced at 30 days after fructose discontinuation (*p* < 0.05) (Figures 6(f) and 6(l)) and were in midterm levels 60 days after fructose discontinuation. The relative visceral fat mass and adipocyte size remained similar between the F and FR groups (Table 3, Suppl. Figures  4(a), 4(b)). Altogether, these data demonstrate the ability of an organism to recover, at least in part, to its previous metabolic pattern, as observed before fructose ingestion.

## 4. Discussion

Rodent models of fructose intake are widely used to mimic (totally or in part) metabolic syndrome characteristics (e.g., increased visceral adiposity, increased circulating triacylglycerol, increased glycemia, reduced insulin sensitivity, and elevated blood pressure) [4, 14]. The development of these main metabolic alterations depends on several aspects including sex [12], animal age at the introduction of fructose [5, 6, 11, 35], the species (rat versus mice) [4, 6, 7, 9–11, 36, 37] (Wistar versus Sprague Dawley rats) [4, 6–14], the quantity [7], and duration [11, 35] of fructose ingested and whether fructose was offered in the tap water or in regular chow [4, 6–9, 11, 12, 22, 37]. Our model reproduced some of the expected alterations, being more pronounced in rats submitted to 30% fructose ingestion, to know (i) increased abdominal fat, (ii) increased triacylglyceridemia, and (iii) increased insulinemia. The supplementation with fish oil for 33 days did not effectively attenuate the elevation in circulating triacylglycerol, but the interruption of fructose ingestion resulted in a better control of this parameter.

The general parameters related to ingestion (water, food, and energy ingestions) in our fructose model were in accordance with previous publications using rats [4, 5, 7, 10, 12]. Male and female rats drinking water containing lower fructose concentrations (commonly 10% fructose from 2 to 8 weeks) ingested higher volumes of water (due to elevated palatability) than control animals [12, 38]. This increased fluid ingestion is compensated by an adaptive reduction in food intake, which usually results in no alteration of the overall amount of energy intake and, consequently, on body mass [12, 38]. Indeed, in our study, rats drinking water containing 10% fructose reproduced exactly the same ingestion behaviors (Figures 2(a)–2(d)). When rats have drunk 30% fructose-containing water, they maintained similar body mass and total energy intake as their matched controls. Furthermore, this was followed by a decrease in water and food intake in relation to their controls (Figures 2(e)–2(h)), a behavior already demonstrated in previous studies with higher fructose concentrations (e.g., 25% and 60% fructose in the tap water) [6, 7]. This feedback leading to reduced food ingestion was previously associated with augmented insulinemia and leptinemia, two known anorexic hormones [8, 9, 11, 13, 14, 35]. Although the total energy intake was unaltered comparing fructose (30% group) and control animals during the 9-week treatment in our study, it is important to emphasize that they had the worst qualitative diet composition. The energy from fructose contributed to around 27% and 40% of the total energy consumed by rats of the 10% and 30% fructose groups, respectively (Figures 3(a) and 3(b)). This may have worsened the parameters found in rats from the 30% group, which developed increased visceral adiposity and higher insulinemia levels (Table 1). The higher the amount of fructose ingestion [7] or the longer the fructose ingestion [11], the more pronounced the adverse effects on metabolism. This augmented visceral adiposity in rats from the 30% fructose group was not followed by an increase in fat lipolysis (Table 1) or adipocyte size (Supp. Figure 3). Although we did not measure the circulating free fatty acids (which could corroborate the elucidation of the lipolysis data) we cannot rule out the possibility that other fat depots than epididymal (e.g., omental, retroperitoneal) could be more lipolytic or that with the prolongation of fructose ingestion the adipose tissue could become more lipolytic. Of note, the increase in visceral adiposity in rats from the 30% fructose group was achieved by the sum of all fat depots (omental, retroperitoneal, and epididymal).

None of the ingestion parameters (e.g., water, food, and energy) were modified by the introduction of fish oil supplementation (Figures 2(a)–2(h)), but the removal of fructose efficiently reversed all of them (water, food, and energy intake). We cannot affirm that these reversions were equivalent to control animals since we did not have a parallel control group. However, by comparing the values for 30 and 60 days' endpoints with the values from day 0 (the last day of fructose ingestion), there is no doubt that the cessation of the treatment was an asset (Figures 6(a)–6(d) and 6(g)–6(j)). Considering that the elevation in circulating insulin and leptin depends on fructose ingestion, the removal of fructose could lead to reductions in both plasma insulin and leptin levels (leptinemia was not quantified in our study).

No major impact of both experimental designs (10% and 30% fructose ingestion) was observed on glucose homeostasis (e.g., basal glycemia, glucose tolerance, and insulin sensitivity based on ITT) (Figures 4(a), 4(d), and 5(a)–5(h)). The main alteration we found was an elevation in circulating triacylglycerol in both designs (10% and 30% groups), which was not robustly persistent until the end of the experiment (Figures 4(b), 4(c), 4(e), and 4(f)). We suggest that rat's age (young rats when the fructose is introduced) and organism plasticity (physiological adaptation to the insult) may be beyond these mild effects of fructose ingestion on such metabolic parameters. There are a number of studies in rodents with fructose intake failing to achieve alterations in parameters related to glucose metabolism [5, 6, 8, 12, 35] and also studies failing to achieve alterations in lipid metabolism [11, 35, 37], which turn this model somehow debatable. As suggested before, one should have two contexts in mind: firstly, the fact that rats were very young (30 days old) at the beginning of our study might have contributed to an elevated capacity to metabolize substrates [39, 40]. The vast majority of rodents' studies with fructose ingestion were done with adult animals [4, 7–14]. Secondly, the organism plasticity could be part of the process in a way that rats overpass fructose adverse effects through adaptive metabolic compensations. In fact, a study similar to ours, with the introduction of fructose ingestion to 21-day-old male rats, demonstrated no alteration in body mass and blood glucose levels with a reduction in food intake and elevation in plasma triacylglycerol (likely in our model) [6]. In addition, alterations such as increased energy intake, relative liver, and visceral adipose tissue masses were observed (unlikely in our model). However, these rats ingested water containing 60% fructose (6-fold and 2-fold higher than our 10% and 30% groups, resp.). Accordingly, it is common to find studies with humans and rats exposed to an intervention causing metabolic alterations over the course of treatment that are not permanent during the follow-up [25, 41]. Such studies reveal the ability of an organism to somehow adapt to these insults.

The absence of effect of fish oil on glucose tolerance and insulin sensitivity in the FFO groups was not unexpected since both F groups did not present any alteration in these parameters. Indeed, fish oil supplementation per se has no impact on normotolerant and insulin-sensitive Wistar rats as previously demonstrated [42]. However, our model was not able to reproduce the antilipidemic action of fish oil supplementation [21, 22] in the FFO groups. It is important to highlight that we began to supplement our rats 30 days after the initiation of fructose ingestion. The studies showing the beneficial action of fish oil supplementation on a fructose-induced elevation in circulating triacylglycerol diverge from ours in some aspects: one is related to the period when the supplementation begins; if it is concomitant with sugar intake the benefit seemed to be more feasible [23, 43]. In addition, when supplementation was introduced after fructose or sucrose intake, fish oil was offered mixed in the solid diet [21, 22]. Although we did not observe an antilipidemic effect of fish oil, we cannot exclude that omega-3 fatty acids were incorporated into esterified lipids, which could be seen as beneficial and that a more prolonged treatment could be effective. Thus, the reproducibility of fish oil antilipidemic action in fructose models merits continuous investigation.

In the present study, F groups exhibited higher levels of fasting plasma insulin than their controls, which occurred concomitantly with unaltered insulin sensitivity (based on ITT) (Table 1). Nevertheless, fish oil exerted no effect on that. Several studies demonstrated that fructose ingestion led to increased plasma insulin values [4, 9–11, 13, 44, 45], while others did not observe this alteration [8, 12, 43]. Many of these studies attributed a reduction of insulin sensitivity based on the HOMA calculus or simply on elevated plasma insulin levels, but no additional tests such as ITT or euglycemic-hyperinsulinemic [9, 13] were applied. Elevation of fasting insulinemia without a reduction in insulin sensitivity in our F groups may be explained by the reduction of insulin-degrading enzyme (IDE) content and activity in the liver. IDE promotes the hepatic insulin clearance, and there are studies with rats ingesting fructose (10% or 20% in tap water) showing a reduction in the IDE protein abundance or mRNA expression both in the cortex and hippocampus [44] and in the liver [45], respectively. This information is important because this context dissociates fructose-induced hyperinsulinemia from peripheral insulin resistance when there is no confirmation. It is important to highlight that we did not find hepatic fat accumulation in 9 out 10 rats drinking fructose (30% group) (Supp. Figure 2), which implies that liver seemed to remain insulin responsive, a question that can be aggravated with the continuation of fructose ingestion.

The main relevance coming from our study resides in the data from our FR groups (both 10% and 30% fructose designs). It was clear that the removal of fructose recovered all parameters previously altered by fructose ingestion to know water and food intake and circulating triacylglycerol (Figures 6(a)–6(l)). Glucose tolerance and insulin sensitivity were not evaluated in our FR groups considering that these parameters were not altered at the end of treatments in the F groups (after 9-week treatment). In a similar study design, mice ingesting corticosterone in the drinking water to induce metabolic syndrome-like phenotype had several metabolic alterations recovered after the removal of glucocorticoid from the tap water [46]. Data from our laboratory (dos Santos et al.,* unpublished results*) indicates that plasma triacylglycerol values remain normalized up to 90 days (where the study was ended) after cessation of combined administration of fructose and glucocorticoid intake. These observations resemble the pregnancy context, where the pregnant organisms are subjected to several adaptive compensations (e.g., increased pancreatic islet mass, mammary gland development, increased triacylglycerol levels, etc.) that are all reversed to the prepregnant period after delivery [47, 48]. Thus, removal of causal factors seems to be promising and merits to be explored in contexts of consolidated metabolic dysfunctions.

In summary, fructose ingestion, beginning early in life, had no major impact but promoted an elevation in the circulating triacylglycerol levels in adult male rats. The effects of fructose ingestion were more pronounced in rats drinking water containing 30% fructose as observed by higher visceral adiposity and insulinemia values. Fish oil supplementation, introduced 30 days after the initiation of fructose ingestion, did not effectively attenuate the elevation in the circulating triacylglycerol and insulin levels caused by fructose ingestion. However, the removal of fructose from drinking water was effective in recovering those parameters altered by fructose ingestion. Thus, we conclude that the interruption of fructose consumption in adult rats is more effective than fish oil supplementation for the recovering of metabolic homeostasis and reinforces that it is never too late to repair improper dietary habits.

## Supplementary Material

Supplemental figure 1: Growth rate. The average normalized food intake (a) and growth rate (b,c) in rats drinking water enriched with 10% fructose (w/v) (a,b) and 30% fructose (w/v) (c). Fructose ingestion lasted 9 consecutive weeks, and fish oil supplementation (1 g/Kg, b.m.) was introduced 30 days after the initiation of fructose ingestion and remained until the end of the ninth week. Results are expressed as mean ± SEM. Asterisk (∗) indicates significantly different compared with the respective control groups (the C and FO groups) using ANOVA with Tukey's post-hoc test (p<0.05, n=10-20). b.m.; body mass.
Supplemental figure 2: Liver histology. The panoramic 10X objective (a,c,e,g,i,k,m) and detailed 40X objective (b,d,f,h,j,l,n) representative images from all groups (only in the experimental design with 30 % fructose ingestion). Only one liver section from the F group (g,h) and one from the FR group (m,n) presented signs of fat accumulation, that were less pronounced in the FR group. Sections were stained with hematoxylin/eosin and mounted with entellan. The images were acquired with a CCD camera coupled to a BX-60 Olympus photomicroscope.
Supplemental figure 3: Adipocyte size after ingestion of 30% fructose. The panoramic 10X objective (a) representative images from all groups (only in the experimental design with 30 % fructose ingestion). Sections were stained with hematoxylin/eosin and mounted with entellan. The images were acquired with a CCD camera coupled to a BX-60 Olympus photomicroscope. The adipocyte area distribution per range (b) and mean adipocyte area (c). Fructose ingestion lasted 9 consecutive weeks and fish oil supplementation (1 g/Kg, b.m.) was introduced 30 days after the initiation of fructose ingestion and remained until the end of ninth week. Results are expressed as mean ± SEM. (n=6).
Supplemental figure 4: Adipocyte size after discontinuation of 30% fructose ingestion. The adipocyte area distribution per range (a) and mean adipocyte area (b). Fructose ingestion lasted 9 consecutive weeks and fish oil supplementation (1 g/Kg, b.m.) was introduced 30 days after the initiation of fructose ingestion and remained until the end of ninth week. Results are expressed as mean ± SEM. (n=6).

## Figures and Tables

**Table 1:** Constitution of commercial fish oil (Phytomare).

	Fatty acids in fish oil	Constitution (%)
*Omega-3*	Eicosapentaenoic acid, EPA	25.3
	Docosahexaenoic acid, DHA	14.8
	Alpha-linolenic acid	0.2

*Omega-6*	Linoleic acid	3.3
	Arachidonic acid	0.1

*Omega-7*	Palmitoleic acid	13.0

*Omega-9*	Oleic acid	9.7

*Saturated fatty acids*	Palmitic acid	18.9
	Myristic acid	9.3
	Stearic acid	5.5

Mean of triplicate. Quantification was done by HPLC.

**Table 2:** Plasmatic, hepatic, and fat data at the day of euthanasia.

	10% fructose				30% fructose			
	C	FO	F	FFO	C	FO	F	FFO
^*1*^ *Insulinemia*	63 [51; 80]	—	144 [99; 191]^*∗*^	133 [117; 164]	62 [51; 89]	—	286 [150; 464]^*∗*^	336 [176; 655]
^*2*^ *Urecemia*	2.0 ± 0.2	1.7 ± 0.3	2.7 ± 0.4	2.3 ± 0.4	1.7 ± 0.2	1.7 ± 0.3	1.6 ± 0.2	2.0 ± 0.4

^*3*^ *Hepatic triacylglycerol content*	2.3 ± 0.7	3.0 ± 0.4	2.5 ± 0.2	3.1 ± 0.3	3.2 ± 0.3	3.4 ± 0.5	3.7 ± 0.2	3.1 ± 0.3

^*4*^ *Basal glycerol release *	7 [5; 8]	7 [6; 13]	6 [5; 8]	8 [6; 10]	5 [3; 6]	3 [2; 5]	6 [5; 7]	5 [4; 6]
^*4*^ *Stimulated glycerol release*	14 [11; 16]	15 [9; 21]	14 [9; 20]	18 [14; 44]	13 [9; 15]	15 [11; 16]	16 [13; 20]	12 [10; 16]

^*5*^ *Visceral fat*	2.6 ± 0.2	2.7 ± 0.2	3.1 ± 0.3	3.5 ± 0.3	2.4 ± 0.2	3.1 ± 0.4	3.7 ± 0.2^*∗*^	3.7 ± 0.3

^*6*^ *TyG index *	8.4 ± 0.07	8.3 ± 0.07	8.5 ± 0.07	8.3 ± 0.06	8.1 ± 0.09	7.8 ± 0.1	8.4 ± 0.04^*∗*^	8.3 ± 0.05

^1^pg/mL; ^2^mg/dL; ^3^mg/g tissue; ^4^*µ*/g tissue·h^−1^; ^5^g/100 g b.m.; ^6^read methods for details. Relative liver, pancreas, and adrenals masses did not change (data not shown). Results are expressed as mean ± SEM for 2,3, 5,6 and median ± interquartile range for 1,4. Asterisk (*∗*) indicates significant difference compared to the C group using ANOVA with Tukey's post hoc test for 5,6 and Kruskal-Wallis with Dunn's post hoc test for 1 (*p* < 0.05, *n = *8–20).

**Table 3:** Visceral fat after interruption of fructose administration (day of euthanasia).

	10% fructose		30% fructose	
	F	FR	F	FR
*Visceral fat (g/100 g b.m.)*	3.1 ± 0.3	3.7 ± 0.4	3.7 ± 0.2	3.8 ± 0.2

Results are expressed as mean ± SEM. ANOVA with Tukey's post hoc test was applied and no differences among groups were achieved (*n* = 20 for the F group and 10 for the FR group).
