# Supplementary material for: Impact of Fish Oil Supplementation and Interruption of Fructose Ingestion on Glucose and Lipid Homeostasis of Rats Drinking Different Concentrations of Fructose
Source: Biomed Res Int. 2017 Aug 8;2017:4378328. doi: 10.1155/2017/4378328 (PMC5591931; doi:10.1155/2017/4378328)
Supplement: Supplementary file 1 — Supplemental figure 1: Growth rate. The average normalized food intake (a) and growth rate (b,c) in rats drinking water enriched with 10% fructose (w/v) (a,b) and 30% fructose (w/v) (c). Fructose ingestion lasted 9 consecutive weeks, and fish oil supplementation (1 g/Kg, b.m.) was introduced 30 days after the initiation of fructose ingestion and remained until the end of the ninth week. Results are expressed as mean ± SEM. Asterisk (∗) indicates significantly different compared with the respective control groups (the C and FO groups) using ANOVA with Tukey's post-hoc test (p<0.05, n=10-20). b.m.; body mass. Supplemental figure 2: Liver histology. The panoramic 10X objective (a,c,e,g,i,k,m) and detailed 40X objective (b,d,f,h,j,l,n) representative images from all groups (only in the experimental design with 30 % fructose ingestion). Only one liver section from the F group (g,h) and one from the FR group (m,n) presented signs of fat accumulation, that were less pronounced in the FR group. Sections were stained with hematoxylin/eosin and mounted with entellan. The images were acquired with a CCD camera coupled to a BX-60 Olympus photomicroscope. Supplemental figure 3: Adipocyte size after ingestion of 30% fructose. The panoramic 10X objective (a) representative images from all groups (only in the experimental design with 30 % fructose ingestion). Sections were stained with hematoxylin/eosin and mounted with entellan. The images were acquired with a CCD camera coupled to a BX-60 Olympus photomicroscope. The adipocyte area distribution per range (b) and mean adipocyte area (c). Fructose ingestion lasted 9 consecutive weeks and fish oil supplementation (1 g/Kg, b.m.) was introduced 30 days after the initiation of fructose ingestion and remained until the end of ninth week. Results are expressed as mean ± SEM. (n=6). Supplemental figure 4: Adipocyte size after discontinuation of 30% fructose ingestion. The adipocyte area distribution per range (a) and mean adipocyte area ( [file 4378328.f1.pdf]

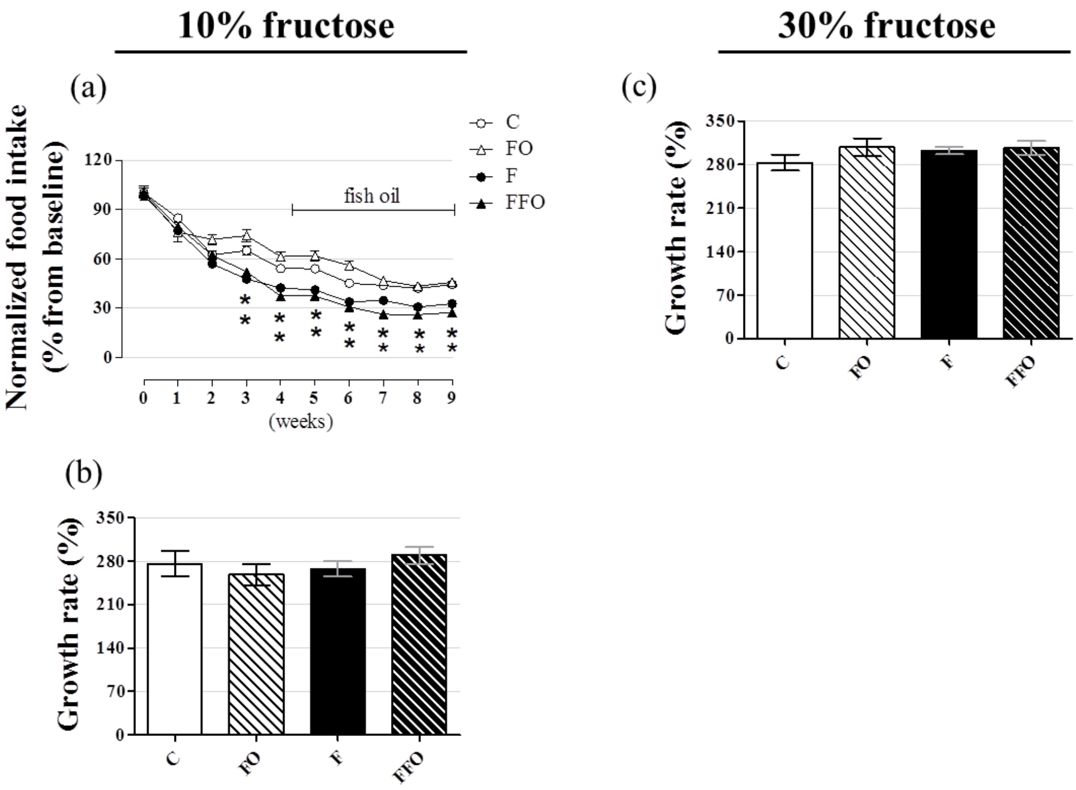

Supplemental figure 1: *Growth rate.* The average normalized food intake (a) and growth rate (b,c) in rats drinking water enriched with 10% fructose (w/v) (a,b) and 30% fructose (w/v) (c). Fructose ingestion lasted 9 consecutive weeks, and fish oil supplementation (1 g/Kg, *b.m.*) was introduced 30 days after the initiation of fructose ingestion and remained until the end of the ninth week. Results are expressed as mean ± SEM. Asterisk (*) indicates significantly different compared with the respective control groups (the C and FO groups) using ANOVA with Tukey's *post-hoc* test ($p<0.05$, $n=10\text{-}20$). ***b.m.**;* body mass.

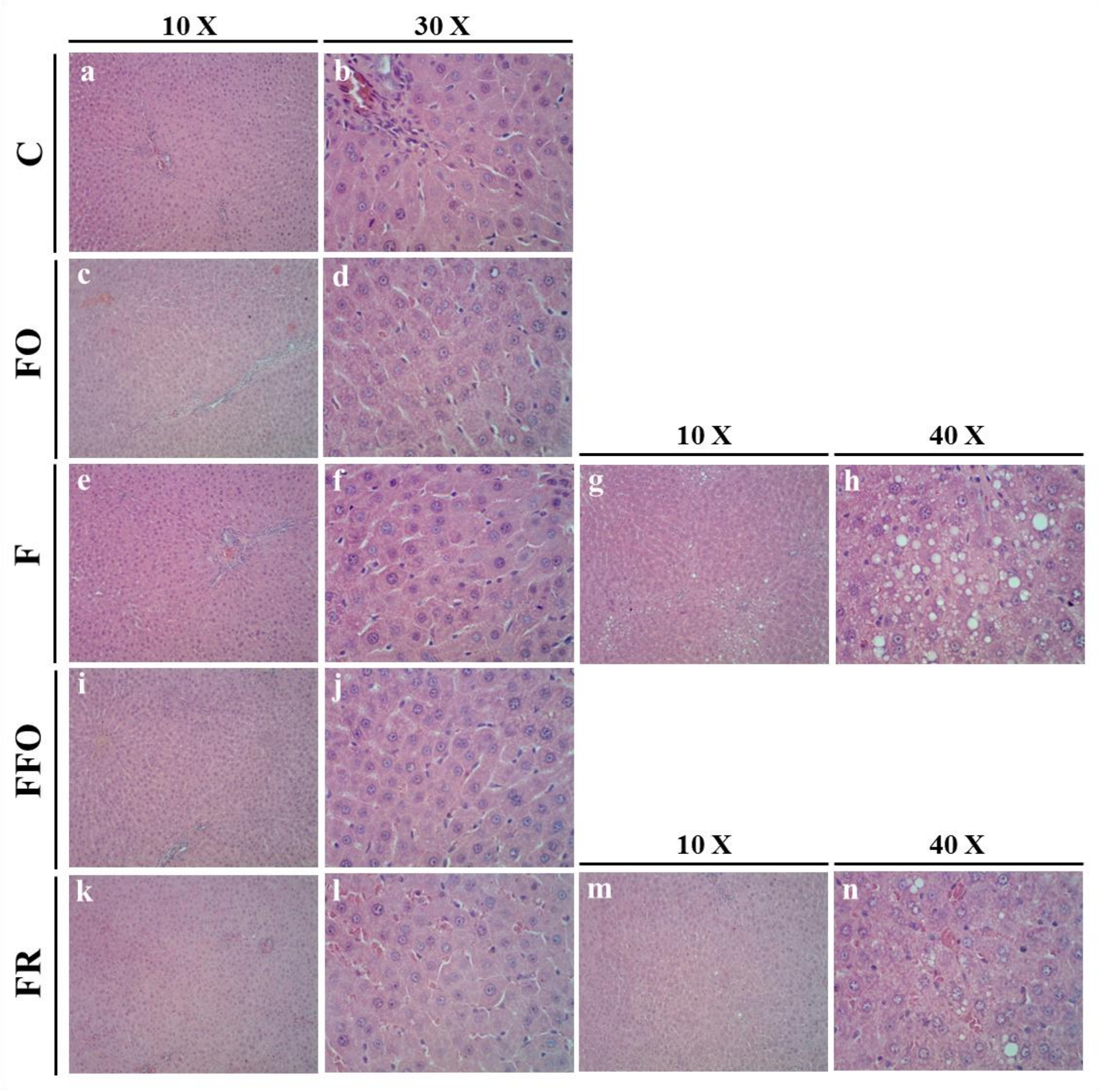

Supplemental figure 2: *Liver histology*. The panoramic 10X objective (a,c,e,g,i,k,m) and detailed 40X objective (b,d,f,h,j,l,n) representative images from all groups (only in the experimental design with 30 % fructose ingestion). Only one liver section from the F group (g,h) and one from the FR group (m,n) presented signs of fat accumulation, that were less pronounced in the FR group. Sections were stained with hematoxylin/eosin and mounted with entellan. The images were acquired with a CCD camera coupled to a BX-60 Olympus photomicroscope.

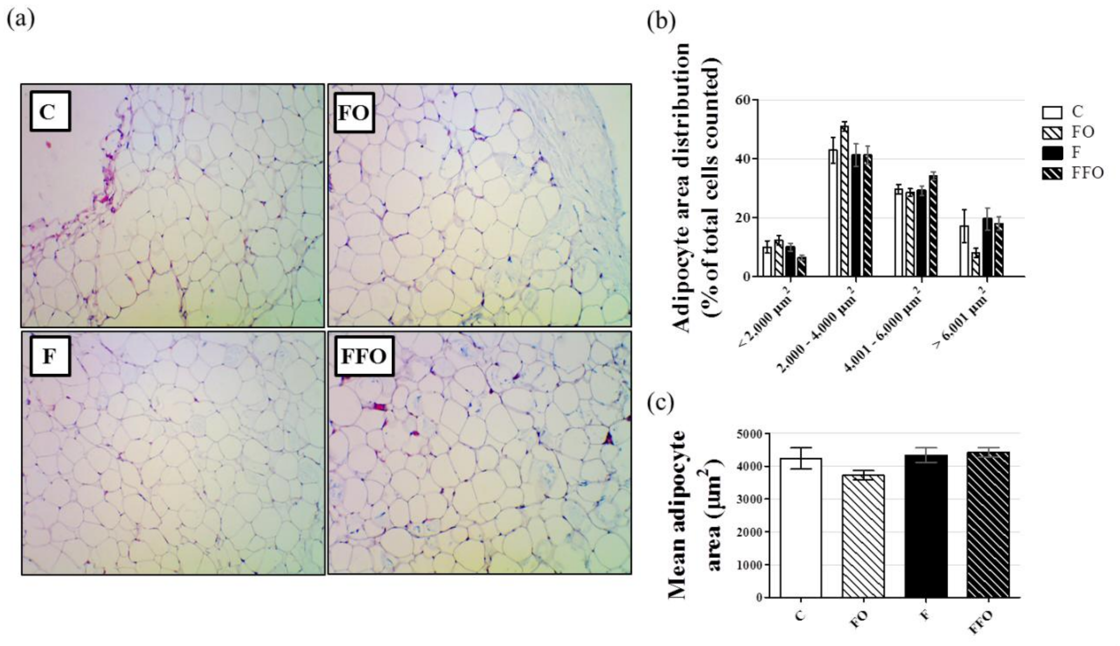

Supplemental figure 3: *Adipocyte size after ingestion of 30% fructose.* The panoramic 10X objective (a) representative images from all groups (only in the experimental design with 30 % fructose ingestion). Sections were stained with hematoxylin/eosin and mounted with entellan. The images were acquired with a CCD camera coupled to a BX-60 Olympus photomicroscope. The adipocyte area distribution *per* range (b) and mean adipocyte area (c). Fructose ingestion lasted 9 consecutive weeks and fish oil supplementation (1 g/Kg, *b.m.*) was introduced 30 days after the initiation of fructose ingestion and remained until the end of ninth week. Results are expressed as mean ± SEM. (*n*=6).

(a)

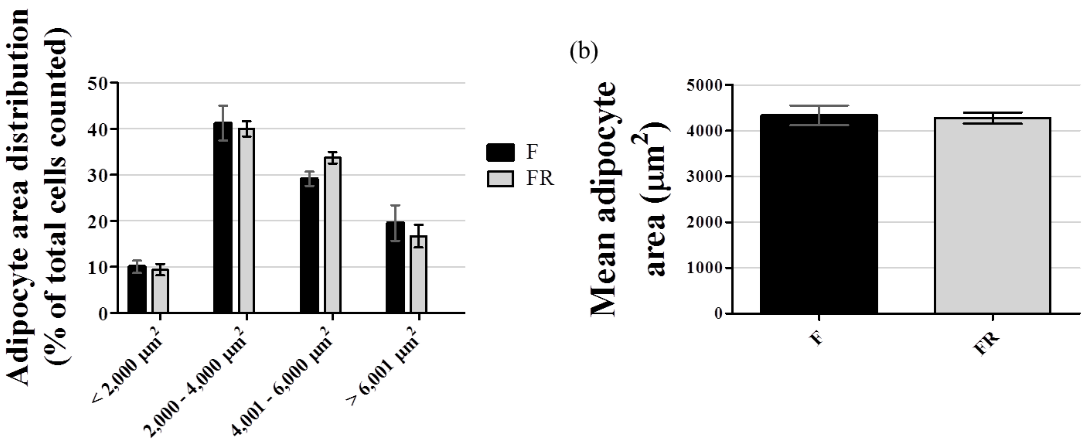

Supplemental figure 4: *Adipocyte size after discontinuation of 30% fructose ingestion*. The adipocyte area distribution *per* range (a) and mean adipocyte area (b). Fructose ingestion lasted 9 consecutive weeks and fish oil supplementation (1 g/Kg, *b.m.*) was introduced 30 days after the initiation of fructose ingestion and remained until the end of ninth week. Results are expressed as mean ± SEM. (n=6).
